# The Controversial Role of Autophagy in Ewing Sarcoma Pathogenesis—Current Treatment Options

**DOI:** 10.3390/biom11030355

**Published:** 2021-02-26

**Authors:** Evangelos Koustas, Panagiotis Sarantis, Michalis V. Karamouzis, Philippe Vielh, Stamatios Theocharis

**Affiliations:** 1 First Department of Pathology, Medical School, National and Kapodistrian University of Athens, 11527 Athens, Greece; vang.koustas@gmail.com (E.K.); psarantis@med.uoa.gr (P.S.); 2 Molecular Oncology Unit, Department of Biological Chemistry, Medical School, National and Kapodistrian University of Athens, 11527 Athens, Greece; mkaramouz@med.uoa.gr; 3 Medipath & American Hospital of Paris, 17 rue Gazan, 75014 Paris, France; ph.vielh@outlook.com

**Keywords:** autophagy, autophagy inducers, autophagy inhibitors, cancer, Ewing Sarcoma

## Abstract

Ewing Sarcoma (ES) is a rare, aggressive, and highly metastasizing cancer in children and young adults. Most ES cases carry the fusion of the Ewing Sarcoma Breakpoint Region 1 (*EWSR1*) and *FLI1* (Friend leukemia virus integration site 1) genes, leading to an EWS–FLI1 fused protein, which is associated with autophagy, a homeostatic and catabolic mechanism under normal and pathological conditions. Following such interesting and controversial data regarding autophagy in ES, many clinical trials using modulators of autophagy are now underway in this field. In the present review, we summarize current data and clinical trials that associate autophagy with ES. In vitro studies highlight the controversial role of autophagy as a tumor promoter or a tumor suppressor mechanism in ES. Clinical and in vitro studies on ES, together with the autophagy modulators, suggest that caution should be adopted in the application of autophagy as a therapeutic target. Monitoring and targeting autophagy in every ES patient could eliminate the need for targeting multiple pathways in order to achieve the maximum beneficial effect. Future studies are required to focus on which ES patients are affected by autophagy modulators in order to provide novel and more efficient therapeutic protocols for patients with ES based on the current autophagy status of the tumors.

## 1. Introduction

Ewing Sarcoma (ES) represents the second most common sarcoma of bone in children. It is a very aggressive and metastatic malignancy, with the most frequent metastatic sites being the lungs and the bone marrow [1]. In patients with localized disease, the five year survival rate is 60–70%, and in those with metastatic disease it is between 20% and 45%. For this reason, new therapeutic approaches are still required in order to enhance the outcome of ES patients, especially those with metastatic disease [2].

The vast majority of ES cases are characterized by the t(11;22)(q24;q12) chromosomal translocation, leading to the fusion of a 5’ segment of the *EWSR1* gene (Ewing Sarcoma breakpoint region 1) and a 3′ portion of the *FLI1* (Friend leukemia virus integration site 1). Furthermore, the fusion between EWSR1 and other members of the ETS family of transcription factors, such as Activating Transcription Factor 1(ATF-1), ETS-related gene (ERG), and Wilms’ tumour 1 (WT1), has been identified in ES [3]. Thus, a pathognomonic chimeric gene is created [4]. The EWS–FLI1 protein increases the deregulation of protein expression by either transcriptionally inducing or repressing specific target genes, many of which are associated with a different oncogenic process, such as cell proliferation, transformation, or tumor growth [5,6,7]. However, the association of EWS–FLI1 with the autophagy process remains unknown. 

EWSR1 is a member of the TET protein family [8]. TET proteins have been identified in many cells and tissues, predominantly repositioned in the nucleus of the cells [9]. In addition, the FET protein family, which includes the EWS, TAF15 (TATA-box binding protein associated factor 15), and FUS/TLS (fused in sarcoma/translocated in liposarcoma, herein referred to as FUS) proteins, has been shown to be a part of the splicing machinery. The three FET proteins are heterogeneously expressed throughout human tissues, with FUS and TAF15 having highly correlated expression patterns. EWS shows cytoplasmic expression in ductal and serous cells but is undetectable in the cytoplasm of mucous cells [10]. Other protein members of the family are FUS and TAF15 [11]. All these proteins have a common structure and function. The TET family member proteins are DNA/RNA-binding proteins (RBPs) with three distinct domains: an N-terminal serine–tyrosine–glycine–glutamine (SYGQ)-rich domain that acts as a transcriptional activation domain; a central RNA recognition domain (RRM); and a C-terminal zinc finger domain, which is associated with sites where TET family proteins bind onto RNA and DNA. [12,13]. The sequences of amino acids in this protein family are highly evolutionarily conserved, share a high homology (~70%) and are evolutionarily conserved from fish to humans [14]. 

In this review, we will try to address autophagy as a key regulator mechanism in ES development and eventually we will suggest this mechanism as a putative chemotherapeutic target for ES. The appearance of new therapeutics targeting relevant pathological processes, the plethora of agents that directly or indirectly modulate autophagy, and the availability of more informative autophagy biomarkers will give us new opportunities for more beneficial therapeutic protocols for ES patients.

## 2. The Complex Mechanism of Autophagy

Autophagy is a basic catabolic mechanism characterized by homeostasis maintenance via the removal of dysfunctional proteins and organelles from the cells [15]. Under normal conditions, cells initiate basal levels of autophagic machinery in order to maintain the homeostasis, biological function, and quality-control of cytoplasmic contents, and to eliminate old, misfolding proteins and damaged organelles [16]. In addition, autophagy represents an essential mechanism that is activated in response to conditions that are too stressful, such as exercise, starvation, and/or immune signaling [16]. During both conditions, “housekeeping” and stress response, cells try to find ways to maintain cytoprotective levels of autophagic machinery while simultaneously avoiding the potentially cytotoxic effects of autophagic activity. This delicate balance implies self-control of autophagy levels and the mechanisms of preventing the degradation and toxification of proteins, cargos, and products [17].

**Figure 1:**
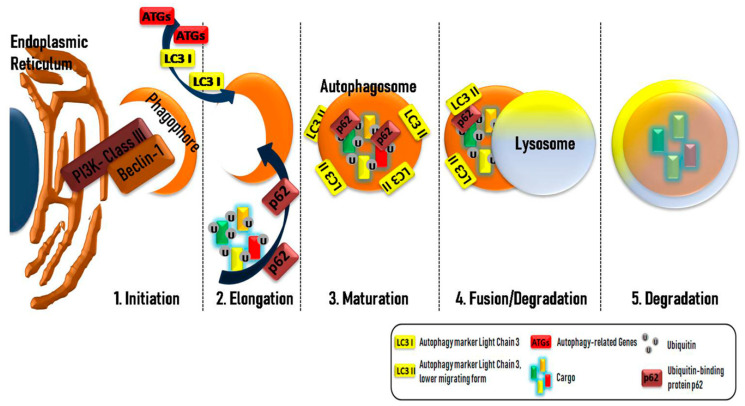
The main steps of autophagosome formation in cells. A plethora of different morphological changes occur during the autophagy process in order to form the autophagosome. The first step, or initiation (1), in this procedure, is the formation of a double-membrane structure, the phagophore, after the activation of a PI3K-classIII–Beclin-1 complex in the endoplasmic reticulum or other double-membrane organelles. Initiation is followed by the elongation (2) step, where the newly formed phagophore begins to enclose the ubiquitin-labeled cytosolic cargos. Different proteins, such as LC3 (light chain 3) (LC3-I is conjugated to phosphatidylethanolamine to form LC3-phosphatidylethanolamine conjugate or LC3-II, responsible for the autophagosomal membrane structure), Atgs (autophagy-related genes), and p62 (an adaptor protein responsible for the docking of cargos), have an essential role in the maturation (3) step, where the autophagosome has already formed. The fusion of the lysosome and autophagosome in the fusion/degradation (4) step creates the autolysosome where, in the degradation step (5), the cytosolic cargos are digested by lysosomal enzymes and the products are released into the cytosol.

Three types of autophagy exist, macroautophagy, microautophagy, and chaperone-mediated autophagy (CMA) [18]. The critical structure of the macroautophagic machinery is the autophagosome [19]. During autophagosome formation, various morphological changes occur. The first step in macroautophagy (hereafter described as “autophagy” unless otherwise mentioned) is the initiation, where a double-membrane structure, the phagophore, is formed through the activation of a complex structure, the class-III PI3K–Beclin-1 complex [20,21]. In elongation (the second step), the phagophore is formed from subcellular membrane structures, such as the Golgi and endoplasmic reticulum (ER), that start to enclose the cytosolic cargos, leading to the formation of the autophagosome. The next step is the maturation of the autophagosome, followed by the fusion with a lysosome (Figure 1). The autolysosome is the structure in which the cytosolic cargo is digested, and the products are released to the cytosol [22].

### 2.1. The Key Components in Autophagy 

A plethora (more than thirty-six) of different autophagy-related (ATG) genes, which play a crucial role in autophagy, have already been characterized [23]. The autophagosome formation is triggered by the ULK-1 and class III PI3K complexes. The critical components in the ULK-1 complex are ATG13, ATG101, ULK1/2, and the family interacting protein FIP200 [24]. mTOR is a main inhibitor of the ULK-1 complex and consequently suppresses autophagy [25,26]. The ULK-1 and class-III PI3K–Beclin-1 complexes, via protein sorting-associated protein 34 (VPS34), are the regulators of autophagy initiation [27,28]. Two ubiquitin-like conjugates, through the interaction of several ATG genes (ATG5-7, ATG10, ATG12, ATG16L1), regulate the elongation of the autophagosome [28]. The second ubiquitin-like pathway is regulated by the interaction of the microtubule-associated protein 1-light chain 3 (LC3-I) with the lipid phosphatidylethanolamine (PE) by ATG3 and ATG7, forming the membrane-bound LC3-II, which is a crucial protein for both sides of the autophagosomal membrane [29,30]. During the maturation step, lysosomal-associated membrane protein 2 and Ras-related protein Rab-7a promote autophagosome fusion with the endocytic and lysosomal compartments in order to form an autolysosome [31,32]. After the formation of the autolysosome [31,32], LC3-II on the cytoplasmic domain of the newly formed organelle (the autolysosome) can be delipidated by ATG4 and recycled. The proteins of the internal surface of the autophagosome are processed for degradation [33] with autophagic cargo through the activity of lysosomal proteases [34]. More detailed guidelines for the selection and interpretation methods for monitoring autophagy are presented in “Guidelines for the use and interpretation of assays for monitoring autophagy (3rd edition)” and can be used by researchers who aim to examine macroautophagy and related processes [35].

### 2.2. The Controversial Role of Autophagy in Human Cancer

The role of autophagy in cell metabolism and tumor growth is controversial [36]. In normal cells, autophagy regulates the energy balance and suppresses carcinogenesis [37]. In contrast, autophagy induces the survival of cancer cells against anti-cancer drugs in already established tumors, and consequently induces tumor growth [38,39]. It appears that anti-cancer therapy (radiotherapy and chemotherapy) induces autophagy as a survival mechanism in cancer cells [40]. Several studies support the hypothesis that radiotherapy initiates autophagy through the up-regulation of several autophagy mediators, such as ATG3-5, ATG12, and Beclin-1. Furthermore, other studies have identified that some chemotherapy agents, such as cisplatin [41] and histone deacetylase (HDAC) inhibitors [42], induce autophagy by increasing the production of ROS in mitochondria. The survival-promoting effect of autophagy has been confirmed by autophagy inhibition [40]. Therefore, the use of autophagy as a putative therapeutic target should be further examined [43].

In the early stage of tumorigenesis, autophagy is characterized as a tumor suppressor mechanism. The first evidence of the association between autophagy and cancer was identified in 1999 when Levine et al. found that Beclin-1 was a candidate for the tumor suppressor gene [44]. The monoallelic deletion of Beclin-1 is detected in breast and ovarian cancer [45]. Furthermore, the overexpression of Beclin-1 decreases the proliferation of colon cancer cell lines [46]. Moreover, Beclin-1 knockout in mice increases the development of spontaneous lymphomas, lung cancers, and liver cancers [47]. Several other components, such as Atg4, Atg5, Atg12, and Atg9b, regulate tumor development [48]. The deletion of Atg7 develops spontaneous benign liver adenomas [49].

In contrast to the early stages of tumorigenesis, autophagy has a beneficial role in already established tumors. Many studies support that autophagy has a crucial role as a survival mechanism under stressful conditions like hypoxia [38]. Under moderate and chronic hypoxia, hypoxia-induced factor-1 (HIF-1a) and PKC-JNK regulate the autophagy levels. A plethora of studies have identified the notion that HIF-1α, which can up-regulate the transcription of Bcl-2/adenovirus E1B 19-kDa-interacting protein 3 (BNIP3), interferes with Beclin-1 and inhibits mTOR. Furthermore, the stress sensor ataxia telangiectasia mutated (ATM) was identified as a modulator of mTOR signaling. It is supported that hypoxia-induced ATM activation leads to an enhancing of the expression of HIF-1α-BNIP3 and Regulated in development and DNA damage response 1 (REDD1) and activates autophagy through the inhibition of the mTOR signaling pathway [50]. Autophagy suppresses hepatocarcinogenesis during the dysplastic stage of the disease and enhances hepatocarcinogenesis in the tumor-forming stage [51]. The dual role of autophagy in cancer is more apparent in colorectal cancer (CRC), as these tumors require high basal levels of autophagy to maintain energy balance, increasing metabolic demands and cell proliferation, especially in hypoxic regions [52].

## 3. The Impact of Autophagy in Ewing Sarcoma 

The controversial role of autophagy as a survival or pro-death mechanism is also identified in different cases of ES. While different mechanisms have been identified to regulate autophagy in ES, it is now well-established that microRNA (miRNA) regulates downstream steps in autophagy, such as initiation, nucleation, elongation, and completion, in different types of cancer [53]. Several studies in this field show that miR125a and miR351 target and destroy *UVRAG* mRNA [54]. It is well known that UV radiation resistance-associated gene (*Uvrag)* regulates autophagy through its interaction with Beclin-1 in order to promote autophagosome formation [55]. In Ewsr1 KO mice, the levels of miR125a and miR351 are increased, a condition that leads to the reduction of UVRAG and LC3II autophagy markers. Besides this, microarray analysis of miRNA has verified that mir125a and miR351 are increased in Ewsr1^−/−^ MEFs and that they depredate *Uvrag* mRNA. This study suggests that EWSR1 regulates autophagy through the epigenetic modulation of UVRAG [54].

Several studies in this field support the hypothesis of autophagy as a protective mechanism under different conditions in ES models. In SK-ES-1 cells (anaplastic osteosarcoma or ES cell line), Beclin-1 knockdown strongly decreased the basic cancer properties of the cell line, such as proliferation, migration, and invasion [56]. Beclin-1 is a crucial regulator for autophagy initiation and its dysfunction has been identified in several cancer types [57]. A silencing of Beclin-1 leads to the inhibition of matrix metallopeptidase (MMP)-9 expressions [56]. In another study, the chimerical protein EWS–FLI1 was shown to trigger autophagy in the NIH3T3 ES cell line. Lu et al. (2017) also showed that EWS–FLI1 proteins interacted with the promoter of the ATG4B gene and, as a result, increased the expression of ATG4B, which is a key regulator of autophagy [58]. Furthermore, it has been shown that EWS–FLI1-dependent autophagy inhibited apoptotic cell death, which was confirmed through the reduction of PUMA and cytosolic cyto-chrome-c, two major pro-apoptotic regulators [59]. Inhibition of autophagy with 3-MA (3-Methyladenine) re-sensitizes the ES cell line to apoptotic cell death [58]. Several studies have already identified the association of NF-kB and autophagy with the regulation of cell death [59,60,61,62]. Studies in ES cell lines showed that treatment with TNF activates NF-kB and mTOR [63], and more specifically, mTORC1 was reported to directly phosphorylate and suppress this kinase complex required to initiate autophagy. Moreover, lacking NF-kB activation, the treatment of ES cell lines with TNF led to autophagy activation through up-regulation of Beclin-1. The authors suggested that autophagy might be a resistance mechanism against anti-cancer therapy in ES. It is well known that mTOR regulates several cellular functions, including autophagy [64]. PTEN (a natural inhibitor of PI3K) deficiency led to the activation of the PI3K/AKT/mTOR signaling pathway [65], which subsequently inhibited apoptosis and increased cell proliferation and anchorage-independent growth [66]. In ES cell lines after the silencing of PTEN, treatment with temsirolimus (a potent mTOR inhibitor) increased autophagy as a protective mechanism under this condition, but the effect of temsirolimus was lost when PTEN was expressed [67]. This study highlighted the complexity of autophagy in cancer, especially in ES. Furthermore, a recent study suggested that TRIM3 (Tripartite Motif Containing 3 inhibits autophagy in Ewing Sarcoma cells. TRIM proteins have been associated with many biological processes, including transcriptional regulation cell differentiation, signaling transduction, and apoptosis. Overexpression of TRIM3 significantly inhibits autophagy through promoting the degradation of Beclin-1, as evidenced by the increases in the amount of P62 (SQSTM1) and the reductions in the amount of LC3B-II, two important markers of autophagy, as well as by the increased LC3 puncta in cells [68]. 

In contrast with the general concept of autophagy as a resistance mechanism in ES, a study in the ES cell lines’ model identified autophagy as a pro-apoptotic mechanism and a possible target for treating ES patients. The anti-cancer agent 2-methoxyestradiol (2-ME) activates apoptotic cell death through the initiation of autophagy in the ES cell line model [69]. In more detail, the authors of this study show that autophagy was activated through the p53-target gene damage-regulated autophagy modulator (DRAM) after treatment with 2-ME in a JNK-dependent manner. The silence of DRAM in ES cells reduced autophagy and apoptotic cell death. Moreover, another study supported that PTEN deficiency led to enhanced AKT activation associated with decreased apoptosis, increased proliferation, and anchorage-independent cell growth. PTEN loss led to increased sensitivity to temsirolimus treatment, as marked by the activation of autophagy [67]. 

## 4. Targeting Autophagy in Clinical Practice—A Promising Anti-Cancer Strategy for Ewing Sarcoma

Following such interesting preclinical data, many clinical trials are now underway in this area. In total, clinical trials using small molecules that directly or indirectly target autophagy have already undergone recruitment, and some of them show encouraging results for ES patients (Table 1). Furthermore, clinical studies on different types of cancer other than ES are presented in Table 1. The combination of the autophagy inhibitor hydroxychloroquine with different drugs (gemcitabine, paclitaxel, carboplatin, etc.) or with mTOR inhibitors (rapamycin, everolimus, temsirolimus) is already used in several types of cancers, such as non-small cell lung cancer (NSCLC), pancreatic cancer, renal cell carcinoma, myeloma and squamous cell cancer (head and neck cancer) (Table 1).

The controversial role of autophagy as a protective or a pro-death mechanism in cancer and tumorigenesis led to the development of molecules that could either inhibit or induce autophagic activity [70,71]. The primary roles of autophagy are to regulate energy balance, remove dysfunctional proteins or organelles, and recycle molecules [72,73]. Thus, it could become the primary target in cancer therapy.

### 4.1. The Clinical Impact of Autophagy Inhibition in Ewing Sarcoma

Because of the vital role of autophagy in different cellular functions, its inhibition should be beneficial as a putative chemotherapeutic strategy, and years of effort led to developing compounds able to inhibit autophagy at different stages (Table 2).

Many studies are already in the clinical phase II using nab-paclitaxel, a molecule that inhibits autophagy through a microtubule stabilizer that inhibits phosphorylation of VPS34 at T159 [74]. One phase II clinical study uses nab-paclitaxel in patients with desmoid tumors (DT) and multiply relapsed/refractory desmoplastic small round cell tumors and ES (ABRADES). The primary goal of this study is to determine the objective response rate (ORR) and the clinical benefit rate (CBR) in subjects with DT, and to determine the objective response rate (ORR) in subjects with desmoplastic small round cell tumor and ES (ClinicalTrials.gov number NCT03275818). In another phase II clinical study, nab-paclitaxel was combined with gemcitabine in order to prevent the formation or growth of tumors in participants’ osteosarcoma, ES, rhabdomyosarcoma, and other soft tissue sarcomas (ClinicalTrials.gov number NCT02945800) (Table 1).

The goal of clinical study NCT01962103 was to find a safe dose of nab-paclitaxel for children with solid tumors (including ES). This study supported that nab-paclitaxel (240 mg/m2 qw3/4) had a tolerable toxicity profile and demonstrated preliminary clinical activity in pediatric patients with solid tumors [75]. A phase II portion of this study evaluating the effect of nab-paclitaxel as a monotherapy for patients with rhabdomyosarcoma, neuroblastoma, and ES is currently enrolling. Another study tried to combine nab-paclitaxel with gemcitabine in patients with pediatric relapsed and refractory solid tumors in order to evaluate if this combinatorial scheme is beneficial for relapsed and/or refractory solid tumors (ClinicalTrials.gov number NCT03507491). Moreover, a phase I clinical study has evaluated the combination of paclitaxel with several other anti-cancer drugs in different types of cancer including ES. The goal of this study was to evaluate the feasibility, toxicity, and maximum tolerated dose of different combinatorial schemes (ClinicalTrials.gov number NCT00002854) (Table 1).

Years of efforts have led to the development of small molecules that inhibit autophagy. The most well-known autophagy inhibitors are the anti-malarial drug chloroquine (CQ) and its derivative hydroxychloroquine (HCQ) which target and inhibit the fusion of lysosomes with autophagosomes [76]. Several clinical trials have already tested the clinical significance of CQ or HCQ, but these trials failed to provide a significant effort due to the lack of consistent autophagy inhibition by these compounds [77]. A plethora of other molecules are already used as autophagy inhibitors, targeting different steps and regulatory mechanisms of autophagy. Such compounds are used as autophagy inhibitors and their main modes of action are presented in Table 2.

### 4.2. The Clinical Impact of Autophagy Induction in Ewing Sarcoma

The association of autophagy with apoptotic cell death, in some cases, led to the development of molecules that induced autophagy (Table 3).

A phase I/II study (NCT03190174) has investigated the combination of nivolumab (an anti-PD-1 monoclonal antibody) with the mTOR inhibitor nab-rapamycin in different sarcoma types, including ES. The primary and secondary objectives of this study were to investigate the maximum tolerated dose of nab-rapamycin and DCR/PFS, respectively, in advanced UPS, LPS, CS, OS, and ES (Table 1).

The mTOR inhibitor everolimus has also been studied with lenvatinib in a clinical study with the ClinicalTrials.gov number NCT03245151. The primary goal of this study was to determine a maximum tolerated dose and a recommended phase II dose, and to describe the toxicities of lenvatinib administered in combination with everolimus in pediatric participants with recurrent/refractory solid tumors. In addition, they tried to estimate the antitumor activity of lenvatinib in combination with everolimus in pediatric participants with selected recurrent/refractory solid tumors, including ES. 

Currently, the study with ClinicalTrials.gov number NCT00949325 shows some encouraging results. The primary purpose of this study was to identify a safe dosing regimen for the combination of temsirolimus and liposomal doxorubicin in patients with recurrent sarcomas. Thus, the combination of the mTOR inhibitor temsirolimus with doxorubicin is safe for heavily pretreated sarcoma patients, and the doxorubicin did not affect the pharmacokinetics of temsirolimus, but it does appear to have increased the exposure to its active metabolite [78]. Furthermore, mTOR inhibition enhances the efficacy of chemotherapy, and re-sensitization may be via sensitizing the chemo-resistant CSC population [79].

Several other molecules are used as autophagy inducers. Rapamycin and rapalogs are the most well-known molecules that induce autophagy through the inhibition of the PI3K/AKT/mTOR signaling pathway. In Table 3, compounds used as autophagy inducers and their primary mechanism of action are shown. 

## 5. Conclusions

The primary role of autophagy has been identified as a highly conservative homeostatic degradation mechanism for dysfunctional cellular organelles and proteins. Autophagic cells capture, depredate, and recycle necessary components in order to maintain the metabolic and energy balance. The vast majority of research studies in the field of autophagy highlight its controversial role as a survival or pro-apoptotic mechanism for different cell types, including cancer ones. The dual role of autophagy is also identified in rare ES tumors. Several in vitro studies on ES linked autophagy with the development and progression of this type of sarcoma. Years of efforts led to the development of many small molecules that directly target autophagy. Currently, a number of clinical trials are underway in this area. In total, clinical trials using small molecules that target autophagy have already undergone recruitment, and some of them show encouraging results for ES patients. Future studies need to focus on the different circumstances in which autophagy is implicated in ES and are expected to provide novel and more efficient therapeutic protocols for patients with ES. Collectively, autophagy appears to have a negative impact on patients’ survival in different types of cancer, including, possibly, ES patients. 

## Figures and Tables

**Table 1:** Clinical studies with autophagy modulators for ES patients.

Number of Study	Intervention/Treatment	Autophagy Modulator and Target	Phase of Study	Other Cancers
NCT03275818	Nab-paclitaxel	nab paclitaxel: Inhibit phosphorylation of VPS34	II	NCT03344172: Pancreatic cancer, gemcitabine, nab-paclitaxel, HCQ and avelumab
NCT02945800	Nab-paclitaxel+gemcitabine	nab paclitaxel: Inhibit phosphorylation of VPS34	II	NCT01649947: NSCLC, Drug: paclitaxel, carboplatin, HCQ, bevacizumab
NCT01962103	Nab-paclitaxel	nab paclitaxel: Inhibit phosphorylation of VPS34	I/II	NCT00728845: NSCLC, Biological: bevacizumab drug: carboplatin, HCQ, paclitaxel
NCT03507491	Nab-paclitaxel+gemcitabine	nab paclitaxel: Inhibit phosphorylation of VPS34	I	
NCT00002854	etoposide, cisplatin, and cyclophosphamide followed by ifosfamide, carboplatin, and paclitaxel	nab paclitaxel: Inhibit phosphorylation of VPS34	I	
NCT03190174	Nab-rapamycin	nab rapamycin: Inhibitor of mTORC1	I	NCT01396200: Myeloma, drug: HCQ, rapamycin
NCT03245151	Everolimus	everolimus: inhibitor of mTORC1	II	NCT01510119: Renal cell carcinoma, Drug: HCQ RAD001 (everolimus)
NCT00949325	Temsirolimus + lip. doxorubicin	temsirolimus: inhibitor of mTORC1	I/II	NCT00909831: Metastatic solid tumors, drug: HCQ, temsirolimus
				NCT01016769: Squamous cell cancer, head and neck cancer, drug: temsirolimus, paclitaxel, carboplatin

NCT: national clinical trial; VPS34: vacuolar protein sorting-associated protein 34; mTORC1: mammalian target of rapamycin complex 1; PI3K Class III: Phosphoinositide 3-kinases (PI3Ks) class III; NSCLC: non-small cell lung cancer; HCQ: hydroxychloroquine.

**Table 2:** Small agents and drugs that effectively inhibit autophagy. In the table, many small molecules and drugs that are already used to inhibit autophagy are shown. Moreover, the main mechanism of action and the target point in the autophagy procedure where they act is shown.

Agents	Mechanism	Target
Chloroquine (CQ)	Neutralizes the acidic pH of intracellular vesicles	Lysosome
Hydroxy-chloroquine (HCQ)	CQ derivative	Lysosome
3-Methyladenine (3-MA)	Inhibitor of PI3K Class I and III	Autophagosome formation
Wortmannin	Inhibitor of PI3K Class I and III	Autophagosome formation
LY294002	PI3-kinase inhibitor	Autophagosome formation
LY3023414	PI3-kinase and mTOR inhibitor	Autophagosome formation
SAR405	(Vps18 and Vps34) inhibitor	Autophagosome formation
SB203580	Inhibitor of p38α and p38β. p38α inhibits trafficking of Atg9	Autophagosome formation
Bafilomycin A1	Inhibition of lysosomal acidification	Lysosome
Concanamycin A	Inhibition of lysosomal acidification	Lysosome
Azithromycin	Inhibition of lysosomal acidification	Lysosome
Paclitaxel	Microtubule stabilizer inhibits phosphorylation of VPS34	Autophagosome formation
SAHA	Inhibit autophagosome–lysosome fusion	Autophagosome formation
Monensin	Inhibit autophagosome–lysosome fusion	Autophagosome formation
Sputin-1	(USP10) and (USP13) inhibitor	Autophagosome formation
NSC185058	Inhibitor of ATG4B	Autophagosome formation
Verteporfin	Alter acidification of lysosomes	Autophagosome formation

PI3K: phosphatidylinositol 3-kinases; VPS: vacuolar protein sorting; ATG: autophagy-related proteins; USP: ubiquitin-specific protease.

**Table 3:** Small agents and drugs that induce autophagy. In the table, molecules and drugs that have been identified as autophagy inducers are shown. Moreover, the main mechanism of action and the target point in the autophagy procedure where they act are shown.

Agents	Mechanism	Target
Rapamycin	Inhibitor of mTORC1	Autophagosome formation
Temsirolimus	Inhibitor of mTORC1	Autophagosome formation
Deforolimus	Inhibitor of mTORC1	Autophagosome formation
Everolimus	Inhibitor of mTORC1	Autophagosome formation
Metformin	AMPK activator	Autophagosome formation
GDC-0980	PI3K and mTORC1 inhibitor	Autophagosome formation
GDC-0941	Inhibitor of PI3K Class I	Autophagosome formation
fluspirilene	Antagonists of L-type Ca^2+^ channels	Lysosome
Perifosine	AKT inhibitior	Autophagosome formation
Tat–Beclin 1 peptide	Releases Beclin-1 into cytoplasm	Autophagosome formation
isoliensinine	Natural alkaloid	Autophagic flux
cepharanthine	Natural alkaloid	Autophagic flux

mTORC1: mammalian target of rapamycin complex 1; AMPK: 5′ AMP-activated protein kinase; PI3K: phosphatidylinositol 3-kinases; AKT: Protein kinase B (PKB); Beclin-1: the mammalian ortholog of the yeast autophagy-related gene 6 (Atg6).

## Data Availability

Data sharing not applicable. No new data were created or analyzed in this study. Data sharing is not applicable to this article.
